# Role of the cGAS/STING pathway in the control of *Brucella abortus* infection acquired through the respiratory route

**DOI:** 10.3389/fimmu.2023.1116811

**Published:** 2023-05-16

**Authors:** Iván M. Alonso Paiva, Raiany A. Santos, Camila B. Brito, Mariana C. Ferrero, Juan Manuel Ortiz Wilczyñski, Eugenio A. Carrera Silva, Sergio C. Oliveira, Pablo C. Baldi

**Affiliations:** ^1^ Cátedra de Inmunología, Facultad de Farmacia y Bioquímica, Universidad de Buenos Aires, Buenos Aires, Argentina; ^2^ Instituto de Estudios de la Inmunidad Humoral (IDEHU), CONICET-Universidad de Buenos Aires, Buenos Aires, Argentina; ^3^ Departamento de Bioquímica e Imunologia, Instituto de Ciências Biológicas, Universidade Federal de Minas Gerais, Belo Horizonte, Minas Gerais, Brazil; ^4^ Laboratorio de Trombosis Experimental, Instituto de Medicina Experimental (IMEX, CONICET-Academia Nacional de Medicina (ANM)), Buenos Aires, Argentina; ^5^ Departamento de Imunologia, Instituto de Ciências Biomédicas, Universidade de São Paulo, São Paulo, Brazil

**Keywords:** *Brucella*, respiratory infection, STING, cGAS, proinflammatory cytokines, innate immunity, protection

## Abstract

Despite the importance of the respiratory route for *Brucella* transmission, the lung immune response to this pathogen is scarcely characterized. We investigated the role of the cGAS/STING pathway of microbial DNA recognition in the control of respiratory *Brucella* infection. After *in vitro B. abortus* infection, CFU numbers were significantly higher in alveolar macrophages (AM) and lung explants from STING KO mice than in samples from wild type (WT) mice, but no difference was observed for cGAS KO samples. CFU were also increased in WT AM and lung epithelial cells preincubated with the STING inhibitor H151. Several proinflammatory cytokines (TNF-α, IL-1β, IL-6, IP-10/CXCL10) were diminished in *Brucella*-infected lung explants and/or AM from STING KO mice and cGAS KO mice. These cytokines were also reduced in infected AM and lung epithelial cells pretreated with H151. After intratracheal infection with *B. abortus*, STING KO mice exhibited increased CFU in lungs, spleen and liver, a reduced expression of IFN-β mRNA in lungs and spleen, and reduced levels of proinflammatory cytokines and chemokines in bronchoalveolar lavage fluid (BALF) and lung homogenates. Increased lung CFU and reduced BALF cytokines were also observed in cGAS KO mice. In summary, the cGAS/STING pathway induces the production of proinflammatory cytokines after respiratory *Brucella* infection, which may contribute to the STING-dependent control of airborne brucellosis.

## 1 Introduction

Brucellosis, a disease produced by Gram-negative bacteria of the *Brucella* genus, is a worldwide-distributed zoonosis. Among the various species included in the genus, *B. abortus*, *B. melitensis* and *B. suis* are the most pathogenic for humans. Domestic and wild animals can be affected, and humans can acquire the infection from several sources, among which contaminated aerosols and food are predominant (1). Given its high infectivity through the respiratory route, *Brucella* has been classified by the CDC as an agent of potential use in bioterrorism (2). Airborne *Brucella* transmission has been linked with several outbreaks of human brucellosis in slaughterhouses, laboratories, and rural areas (3–6). Despite the importance of the respiratory route for *Brucella* transmission, the lung immune response to this pathogen is only partially characterized. Given the ability of this pathogen to disseminate systemically within a few days after respiratory infection, the study of the lung innate immune response to the bacterium is of special interest.

Inhaled microorganisms first contact lung epithelial cells and alveolar macrophages (AM), which can respond to this threat by several mechanisms, including not only macrophage phagocytosis but also the production of proinflammatory factors, antimicrobial peptides and type I interferons (7–9). Previous studies have shown that lung epithelial cells produce proinflammatory factors upon infection with *Brucella abortus* or upon interaction with *Brucella*-infected macrophages (10). AM also produce proinflammatory cytokines in response to *B. abortus* infection (11). These immune responses depend in a great proportion on the recognition of microbial components by innate immunity receptors. The cytokine response of AM to *B. abortus* depends mainly on TLR2 recognition (11). A role for TLR2 and TLR4 in the control of respiratory *Brucella* infection has been shown by some studies (12) (13). Besides TLR, other innate sensors may contribute to induce immune responses to *Brucella* in the lung. Inflammasomes are multimeric complexes located in the cytosol that activate caspase-1 to mediate the cleavage of pro-IL-1β and pro-IL-18 into their mature active forms. It has been shown that some inflammasomes (NLRP3, AIM2) contribute to the control of pulmonary *Brucella* infection, probably through the induction of IL-1β maturation (14). While NLRP3 responds to diverse stimuli that exert cellular perturbations (pathogens, microbial toxins, etc.), AIM2 is a cytosolic sensor capable of detecting double-stranded DNA of microbial or host origin. Besides AIM2, one of the main cytosolic receptors for microbial nucleic acids is STING, although its activation leads to other biological consequences (15). Whereas STING recognizes directly some cyclic dinucleotides produced by invading pathogens, it does not recognize whole DNA. Instead, the cyclic GMP-AMP synthase (cGAS) binds DNA and catalyzes the production of cyclic dinucleotides that are subsequently recognized by STING (16, 17). Upon this sensing STING suffers a conformational change that triggers its association with TANK-binding kinase 1 (TBK1) and the migration of the STING-TBK1 complex from the ER to endosomal/lysosomal perinuclear regions. The translocation of TBK1 induces the phosphorylation of the transcription factors IRF3 and NF-κB. The latter translocate to the nucleus and trigger the transcription of genes coding for type I IFNs and proinflammatory cytokines, respectively (18, 19).

Whereas the STING pathway was initially believed to be only relevant for the immune response against viruses (due to its role in type I IFN production), later studies revealed a role for STING in the control of infections produced by bacteria and protozoans (18, 20, 21). Although cGAS and STING have been shown to be expressed in alveolar macrophages and lung epithelial cells (22–24), the role played be the cGAS/STING pathway in the lung immune response to inhaled *Brucella* is unknown. While a previous study has shown that STING is important in the defense against systemic (intraperitoneal) *B. abortus* infection (25), the role of the cGAS/STING pathway in *Brucella* infections acquired through a natural (mucosal) route has not been explored. This difference is not trivial, as the anatomical barriers and the cellular components involved in the host-pathogen interaction are quite different for both infection routes. In this study we show that the cGAS/STING pathway is involved in the control of *Brucella* infection acquired through the respiratory route in the mouse model. We also show that this pathway mediates the production of proinflammatory cytokines and IFN-β in the lung in response to *B. abortus* infection.

## 2 Materials and methods

### 2.1 Bacterial strains and growth conditions

*B. abortus* 2308 were grown in tryptic soy broth (TSB) at 37°C with agitation grown until the bacterial suspension reached an OD_600_ ≈ 1.0. After centrifugation and washing of the bacterial pellet with sterile phosphate buffered saline (PBS), inocula were prepared by resuspending bacteria to the desired OD_600_. *Brucella* manipulations for both *in vitro* and *in vivo* experiments were conducted under BSL3 conditions.

### 2.2 Mice

Animals for *in vivo* studies were C57BL/6 wild-type mice (6–9 wk of age) and knock-out (KO) mice of the same background (cGAS and STING KO mice), all provided by the Federal University of Minas Gerais (UFMG), Brazil. Both strains of KO mice have been described previously (26, 27). Housing conditions were: 5 animals per cage, controlled temperature (22°C±2°C) and a 12 h cycle period of artificial light. Animals received sterile food and water *ad libitum*, and were maintained under specific pathogen-free conditions in positive-pressure cabinets. C57BL/6 mice for *in vitro* studies (primary cultures) were provided by IMEX, National Academy of Medicine, Buenos Aires, Argentina, and were kept under similar conditions as mentioned above until processing. All animal experiments were conducted according to international ethical standards (Amsterdam Protocol of welfare and animal protection, and National Institutes of Health, USA, guidelines: Guide for the Care and Use of Laboratory Animals), and are reported in accordance with ARRIVE Guidelines. The animal protocols used in this study were approved by the respective Committees for the Care and Use of Experimentation Animals from the UFMG (CETEA no. 69/2020) and the School of Pharmacy and Biochemistry of the University of Buenos Aires (Res. D1879/2019).

### 2.3 Intratracheal inoculation

Intratracheal inoculation of animals with *B. abortus* was performed as previously described (28) with minor modifications (29). Briefly, after receiving isoflurane anesthesia mice were intraperitoneally injected with ketamine/xylazine (100mg/kg and 8mg/kg). Animals were placed over an acrylic backboard in supine position and their head movements were restrained with a rubber band passed under their upper incisors. Under translucent illumination of the trachea each mice was injected in between the vocal cords using a blunt-ended probe with 20 µl of *B. abortus* suspension containing 10^5^ CFU (as estimated by OD_600_).

### 2.4 CFU and cytokine analysis

Colony-forming units (CFU) of *B. abortus* and cytokine levels in homogenates of selected organs from infected mice were determined as described previously (14). Briefly, at different post-infection (p.i.) time-points an intraperitoneal lethal dose of ketamine and xylazine was given to each animal. Their lungs, livers and spleens were homogenized in sterile PBS using a tissue homogenizer (Bio-Gen PRO200™). Some homogenate aliquots were serially diluted and plated on TSA for CFU counting, whereas the remaining homogenate volumes were processed for cytokine measurements. In the later case, samples were centrifuged at 4°C and the supernatants were mixed with protease inhibitors (cOmplete™, Roche) and stored at -70°C. Cytokine and chemokine determinations were performed using commercial ELISA kits (R&D).

### 2.5 Murine alveolar macrophages and broncho-alveolar lavage fluid

Murine alveolar macrophages were isolated following previously described procedures (11). Mice were euthanized with the ketamine/xylazine lethal dose. Their tracheas were exposed and a small incision was performed to insert a fine-tipped pipette. Sterile cold PBS containing 1 mM EDTA was perfused (3 times, 1 ml each) to obtain broncho-alveolar lavage fluid (BALF). After centrifugation (400 x*g*, 10 min, 4°C) BALF supernatants were stored at -70°C for cytokine measurements, whereas pellet cells were resuspended in complete medium (RPMI 1640 containing 10% heat inactivated fetal bovine serum -FBS, Gibco-, 100 U/mL of penicillin and 50 µg/mL of streptomycin). In trypan blue exclusion assays the viability of BALF cells was routinely greater than 95%. After incubation for 2 hours (37 °C, 5% CO_2_ humidified atmosphere) in 48-wells culture plates (2x10^5^ cells/well) to allow adhesion of alveolar macrophages, non-adherent cells were removed by repeated washes with fresh culture medium.

### 2.6 RT-PCR for *IFN-β* expression

The expression of IFN-β in lungs and spleen from mice intratracheally infected with *B. abortus* was evaluated by RT-PCR as previously described (25). Total RNA was extracted from lungs and spleens with TRIzol reagent (Invitrogen) and was quantified with a NanoDrop^®^ spectrophotometer (PeqLab, Erlangen, Germany). The synthesis of cDNA from RNA (1 µg) was carried out in a final reaction volume of 20 µl. The reverse transcription was carried out at 50°C for 30 min and the reaction was stopped by denaturation of the enzyme (85°C, 10 min) using Illustra Ready-To-Go RT-PCR Beads (GE Healthcare) following the manufacturer’s instructions. Real-time RT-PCR was performed on a QuantStudio3 real-time PCR instrument (Applied Biosystems) with 2X SYBR Green PCR master mix (Applied Biosystems). The following primers were used to amplify a specific fragment of specific gene targets: 18S, forward, 5´-CGTTCCACCAACTAAGAACG-3´, reverse, 5´-CTCAACACGGGAAACCTCAC-3´; IFN-β, forward, 5’-GCC TTT GCC ATC CAA GAG ATG C-3’, reverse, 5’-ACA CTG TCT GCT GGT GGA GTT C-3’. The sequence of the 18S primer was previously published (30). For each gene a negative control (NTC, non-template control) was used and the presence of non-specific amplification product was checked by the melting curve. Data were analyzed using the threshold cycle (*ΔΔCT*) method and are informed as relative expression units after normalization to the *18S* gene. The calibrator used were samples from uninfected mice, compared to infected WT and KO animals.

### 2.7 Primary lung epithelial cells

Lung epithelial cells (LEC, mostly type II pneumocytes) were isolated from murine lungs as described previously (14). Briefly, remaining blood in the lungs was eliminated by perfusion through the heart with sterile PBS. After washing with an appropriate buffer, the organs were cut into small pieces which were digested with trypsin at 37°C for 10 min. Filtration through a 100 µm mesh was performed, with the filtrate recovered on FBS containing DNAse. After a second filtration through a 40 µm mesh, the final filtrate (containing individual cells) was centrifuged. The LEC-enriched pellet was resuspended in DMEM/F12 containing DNAse. The cellular suspension was incubated for 2 h in 6-well culture plates, after which the supernatant containing non-adherent cells was subjected to a discontinuous Percoll gradient (1.089 g/l and 1.04 g/l; Sigma). The LEC-enriched interface was recovered and washed twice. An aliquot was taken for viability determination (Trypan blue) and counting. For infection experiments, LEC were resuspended in supplemented DMEM/F12 and seeded at 2×10^5^ cells/well in 48-well plates coated with type I collagen. Culture medium was changed daily until the cells reached confluence (5-7 days).

### 2.8 Cell infections

Alveolar macrophages (AM) and LEC were infected *in vitro* with *B. abortus* at multiplicities of infection (MOI) of 100 bacteria/cell (AM) or 200 bacteria/cell (LEC) as described previously (14). Bacteria were dispensed suspended in culture medium without antibiotics, followed by centrifugation of the plates and incubation for 2 hours (37°C, 5% CO_2_ atmosphere). Bacteria not adhered or internalized into the cells were removed by three washes with sterile PBS (time 0 p.i.). To kill non-internalized bacteria the cells were incubated in culture medium containing 100 µg/ml of gentamicin (Sigma, USA) and 50 µg/ml of streptomycin (Sigma, USA). Culture supernatants were harvested for cytokine measurement 24 h after antibiotics addition. At the same time, cell lysates prepared using 0.2% Triton X100 were plated on TSA to enumerate CFU of intracellular bacteria. In some experiments, a STING inhibitor (H151, InvivoGen, 15 µM) or its vehicle (DMSO) were added to AM and LEC for 24 h before infection, and were maintained during the whole infection period.

### 2.9 Statistical analysis

Data were analyzed using Student's *t* test or analysis of variances (ANOVA). Tukey’s post-test and Dunnett’s post-test were used for multiple comparisons between all pairs of groups and against a control group, respectively. A p<0.05 was considered as statistically significant. All statistical analyses were performed with the GraphPad software (San Diego, CA).

## 3 Results

### 3.1 STING is involved in the control of *Brucella* infection by pulmonary cells

**Figure 1:**
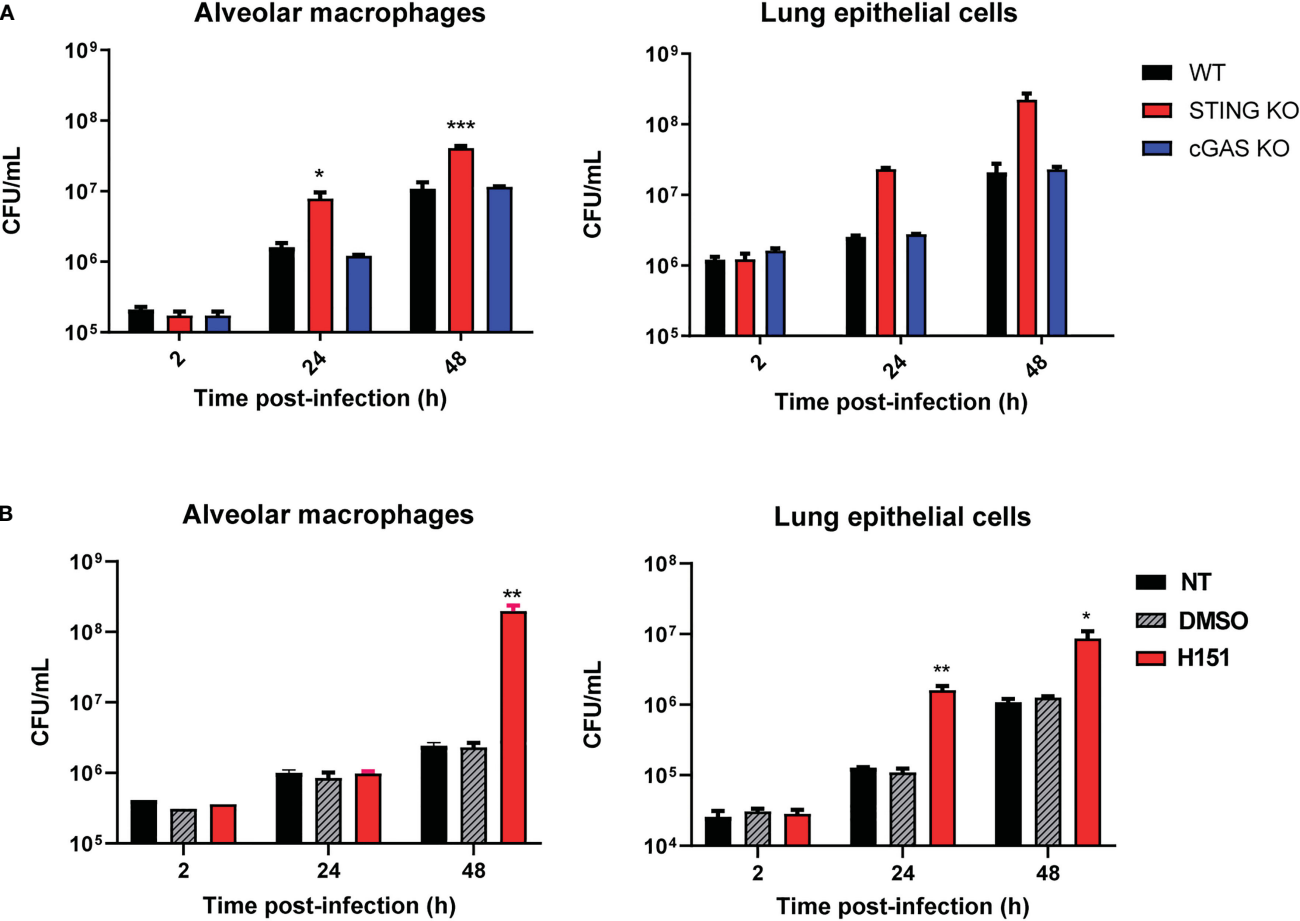
Influence of STING and cGAS on the course of *B. abortus* infection in lung explants and cells infected in vitro. **(A)** Alveolar macrophages (5x10^5^/well) and lung explants (1 cm^3^) of wild type (WT) C57BL/6 mice and STING KO and cGAS KO mice were infected with *B. abortus* 2308, and were processed at different time points to determine intracellular colony-forming units (CFU) (n= 3 per time and mouse strain). **(B)** Alveolar macrophages and lung epithelial cells from C57BL/6 mice were pretreated (H151) or not (NT) with the specific STING inhibitor H151 before infection, and CFU were determined at different post-infection times. Cells pretreated with the vehicle (DMSO) served as controls (n= 3 per time and treatment). Values are means ± SD of two independent experiments (*p<0.05, **p<0.01, ***p<0.001 versus WT **(A)** or INF **(B)**, ANOVA followed by Tukey´s test).

Alveolar macrophages (5x10^5^/well) and lung explants (1 cm^3^) of wild type C57BL/6 mice and STING KO and cGAS KO mice of the same genetic background were infected with *B. abortus* 2308, and were processed at different time points to determine intracellular CFU. At both 24 and 48 h p.i., CFU levels were significantly higher in alveolar macrophages and explants from STING KO mice than in samples from normal mice (**Figure 1A**). In contrast, the bacterial load did not differ significantly between cGAS KO and wild type mice for any sample at any time point. Given this last observation, the ensuing experiments were focused on the role of STING. The involvement of STING in the control of *Brucella* infection by lung cells was confirmed when alveolar macrophages and LEC were pretreated with the specific STING inhibitor H151 before infection. As shown in **Figure 1B**, CFU were significantly increased at 24 and/or 48 h p.i. in cells preincubated with the inhibitor than in untreated cells or those pretreated with vehicle (DMSO).

### 3.2 STING and cGAS mediate the production of proinflammatory cytokines by *Brucella*-infected pulmonary cells

**Figure 2:**
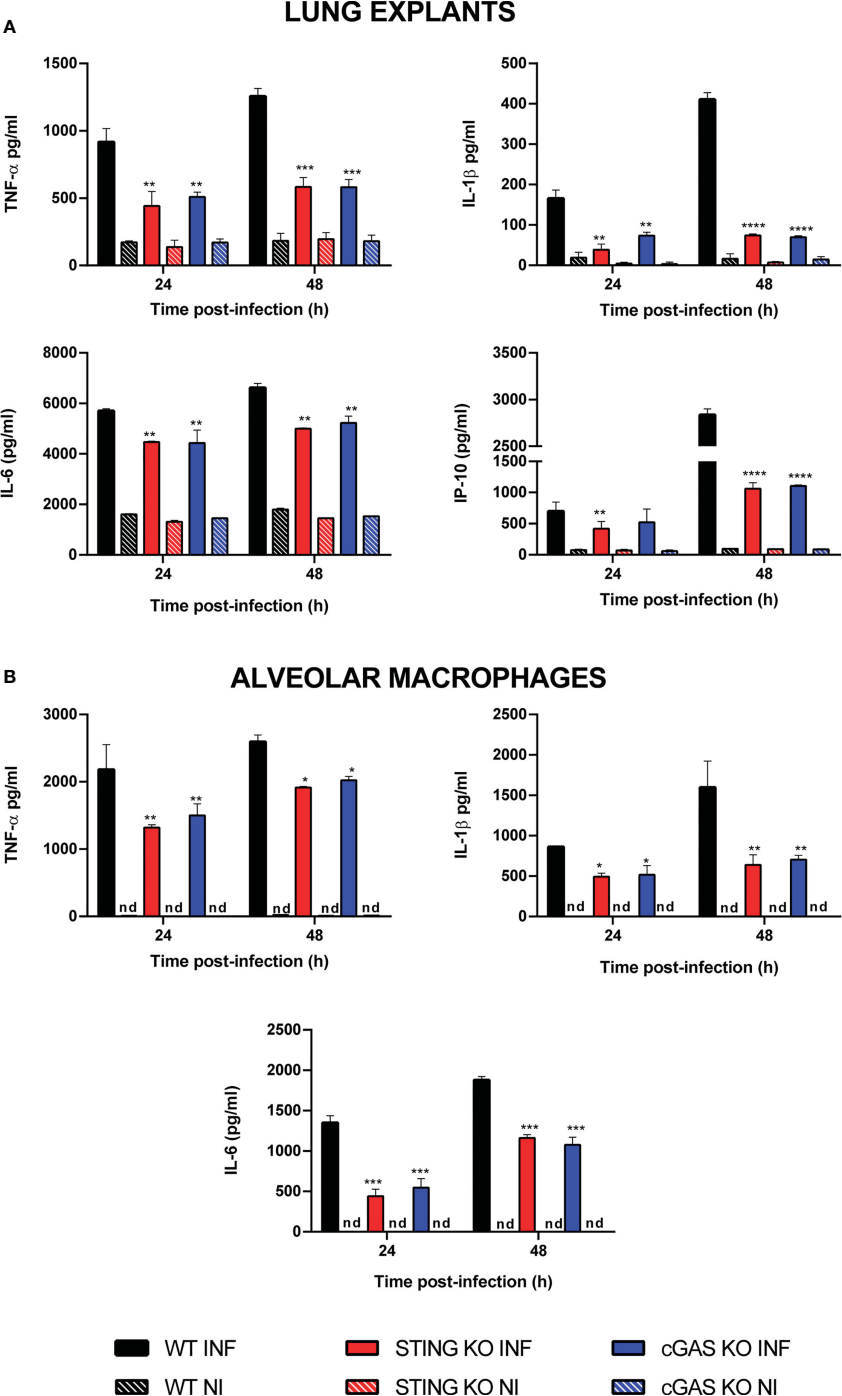
Influence of STING on the production of proinflammatory factors by *Brucella*-infected lung explants **(A)** and alveolar macrophages **(B)**. Explants (1 cm^3^) or alveolar macrophages from wild type (WT) and STING KO C57BL/6 mice were infected in vitro with *B. abortus* 2308, and culture supernatants were collected at 24 and 48 h post-infection to measure cytokine levels by sandwich ELISA (n= 3 per time and mouse strain). Values are means ± SD of two independent experiments (*p<0.05, **p<0.01, ***p<0.001, ****p<0.0001, STING KO versus WT, Student’s *t* test). INF: infected, NI: non-infected, nd: not detected.

**Figure 3:**
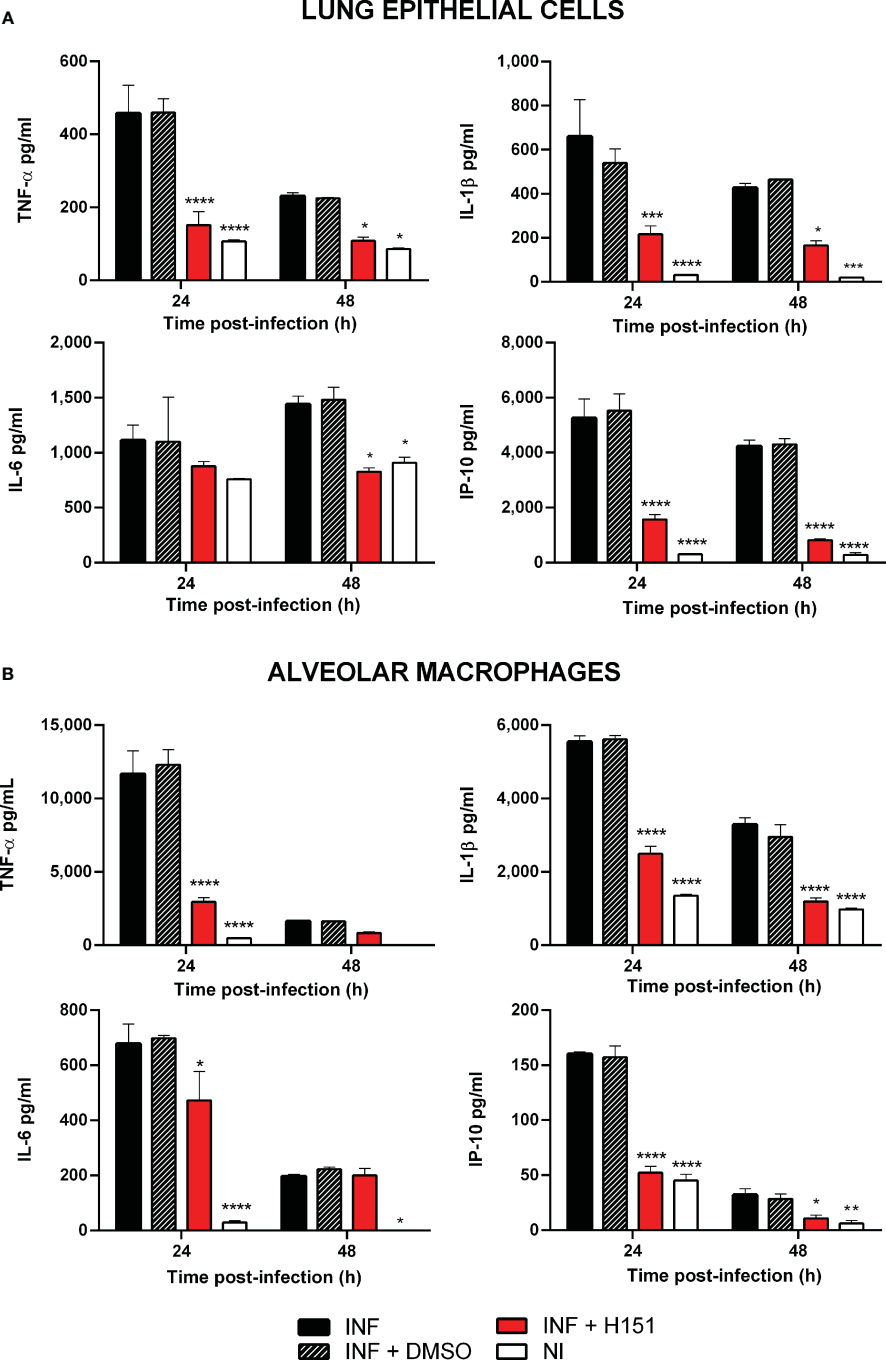
Effect of STING inhibition on the production of proinflammatory factors by *Brucella*-infected lung epithelial cells **(A)** and alveolar macrophages **(B)**. Lung epithelial cells or alveolar macrophages from C57BL/6 mice were pretreated (INF+H151) or not (INF) with the specific STING inhibitor H151 before infection, and culture supernatants were collected at different post-infection times to measure cytokines by sandwich ELISA. Non-infected cells (NI) and cells pretreated with the vehicle (INF+DMSO) served as controls (n= 3 per time and treatment). Values are means ± SD of two independent experiments (*p<0.05, **p<0.01, ***p<0.001, ****p<0.0001 versus INF, ANOVA followed by Tukey´s test).

As mentioned, the cGAS/STING pathway mediates not only the production of type I interferons but also the production of proinflammatory cytokines and chemokines. As shown in **Figure 2A**, the levels of several proinflammatory cytokines (TNF-α, IL-1β, IL-6) and of the proinflammatory chemokine IP-10/CXCL10 were significantly reduced in lung explants from STING KO mice and cGAS KO mice infected *in vitro* with *B. abortus* as compared to explants from wild type mice. Although several cell types can produce proinflammatory mediators in the lung, alveolar macrophages and epithelial cells are especially important as they are the first to contact inhaled pathogens. Therefore, the role of STING and cGAS in the proinflammatory response of these two cell types was investigated. As shown in **Figure 2B**, the secretion of TNF-α, IL-1β and IL-6 in response to *B. abortus* infection was significantly reduced in alveolar macrophages from STING KO mice and cGAS KO mice as compared to wild type mice. In line with these findings, the levels of these cytokines were significantly reduced in LEC (**Figure 3A**) and alveolar macrophages (**Figure 3B**) pretreated with the STING inhibitor H151 than in untreated cells or those pretreated with vehicle (DMSO).

### 3.3 STING is involved in the control of *Brucella* infection acquired through the airways

**Figure 4:**
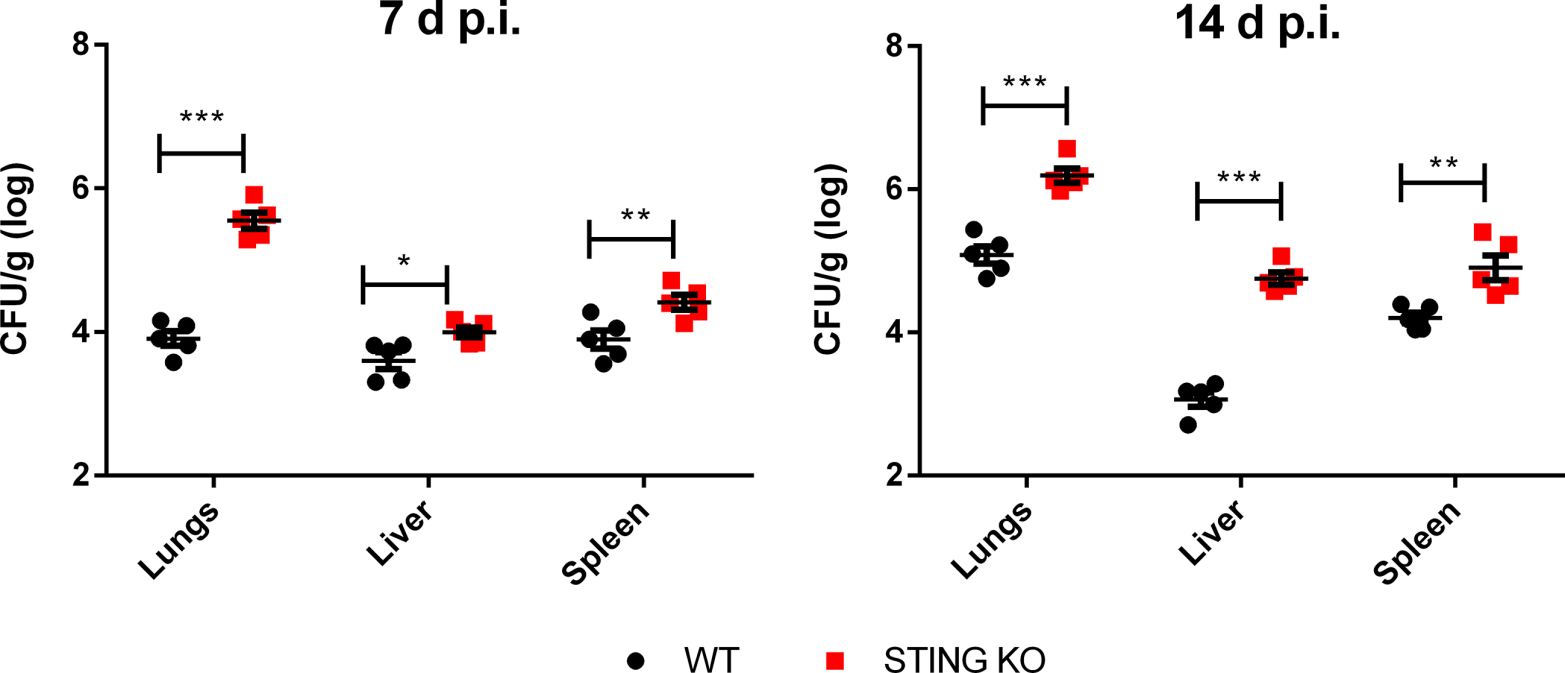
STING is involved in the control of *Brucella* infection acquired through the airways. Wild type (WT) and STING KO mice (n=5 per group) were infected intratracheally with *B. abortus* (10^5^ CFU/mice) and CFU were measured at 7 and 14 days p.i. in lungs and peripheral target organs (spleen and liver). Values are data from a representative experiment of two performed with similar results (*p<0.05, **p<0.01, ***p<0.001, Student’s *t* test).

In view of the role of STING in the control of **in vitro* B. abortus* infection in lung explants, alveolar macrophages and lung epithelial cells, we decided to test the role of this receptor in the control of the *in vivo* respiratory infection. To this end, wild type and STING KO mice were infected intratracheally and CFU were measured at 7 and 14 days p.i. in lungs and peripheral target organs (spleen and liver). As shown in **Figure 4**, at both time points the bacterial load was significantly higher in all the investigated organs in STING KO mice than in wild type mice, revealing that this receptor is involved in the control of respiratory *Brucella* infection.

### 3.4 *Brucella abortus* induces a STING-dependent expression of *IFN-β* in the lungs

**Figure 5:**
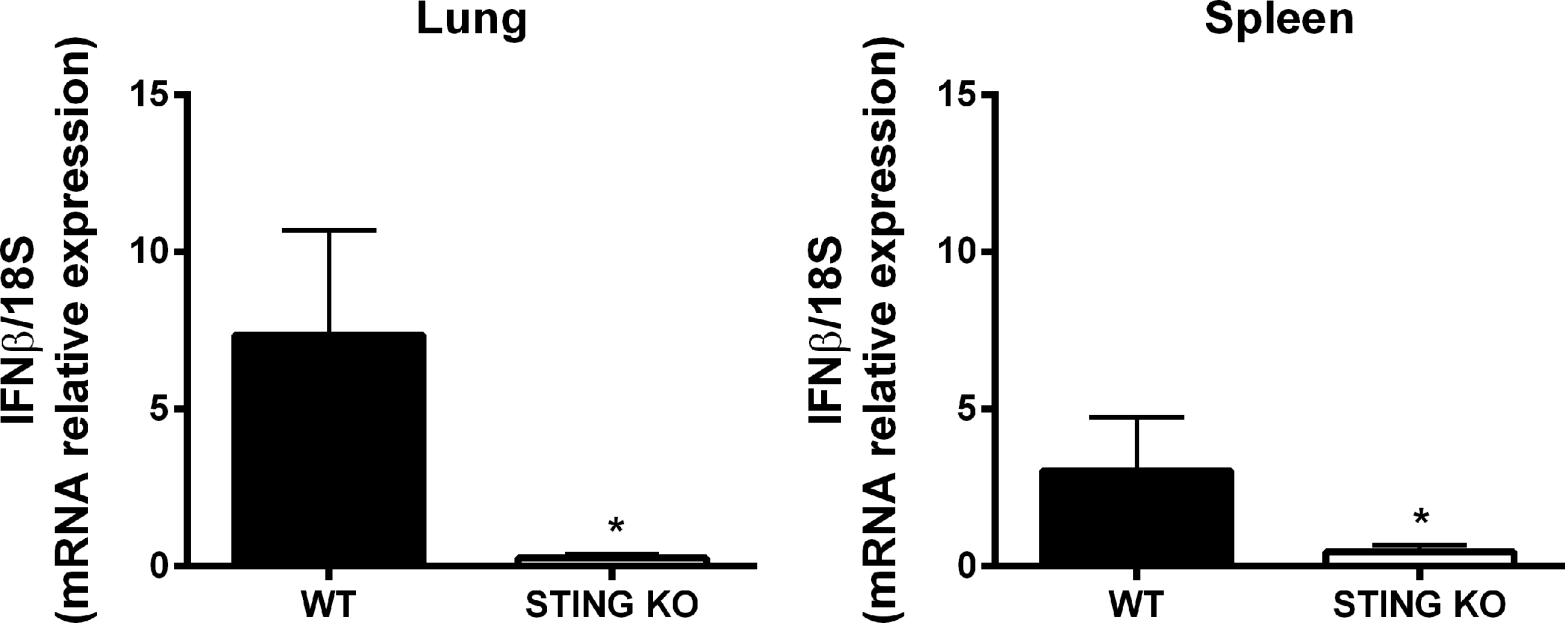
STING-dependent expression of *IFN-β* in mouse organs after intratracheal *B. abortus* infection. Wild type (WT) and STING KO mice (n=5 per group) were infected intratracheally with *B. abortus* (10^5^ CFU/mice) and the expression of *IFN-β* was measured by RT-PCR at 7 days p.i. in lungs and spleen. Values are means ± SD of two independent experiments (*p<0.05, Student’s *t* test).

The STING pathway constitutes a central mediator of type I IFN responses, which have recently been shown to have a role not only in protection against viruses but also against other microorganisms, including some respiratory bacterial pathogens. Therefore, we tested the expression of *IFN-β* mRNA by qRT-PCR in lungs and spleens of mice at 7 days after intratracheal *B. abortus* infection. As shown in **Figure 5**, *IFN-β* expression was significantly reduced in both organs in STING KO mice as compared to wild type mice.

### 3.5 STING mediates the production of proinflammatory cytokines in lungs and peripheral organs after respiratory *Brucella* infection

**Figure 6:**
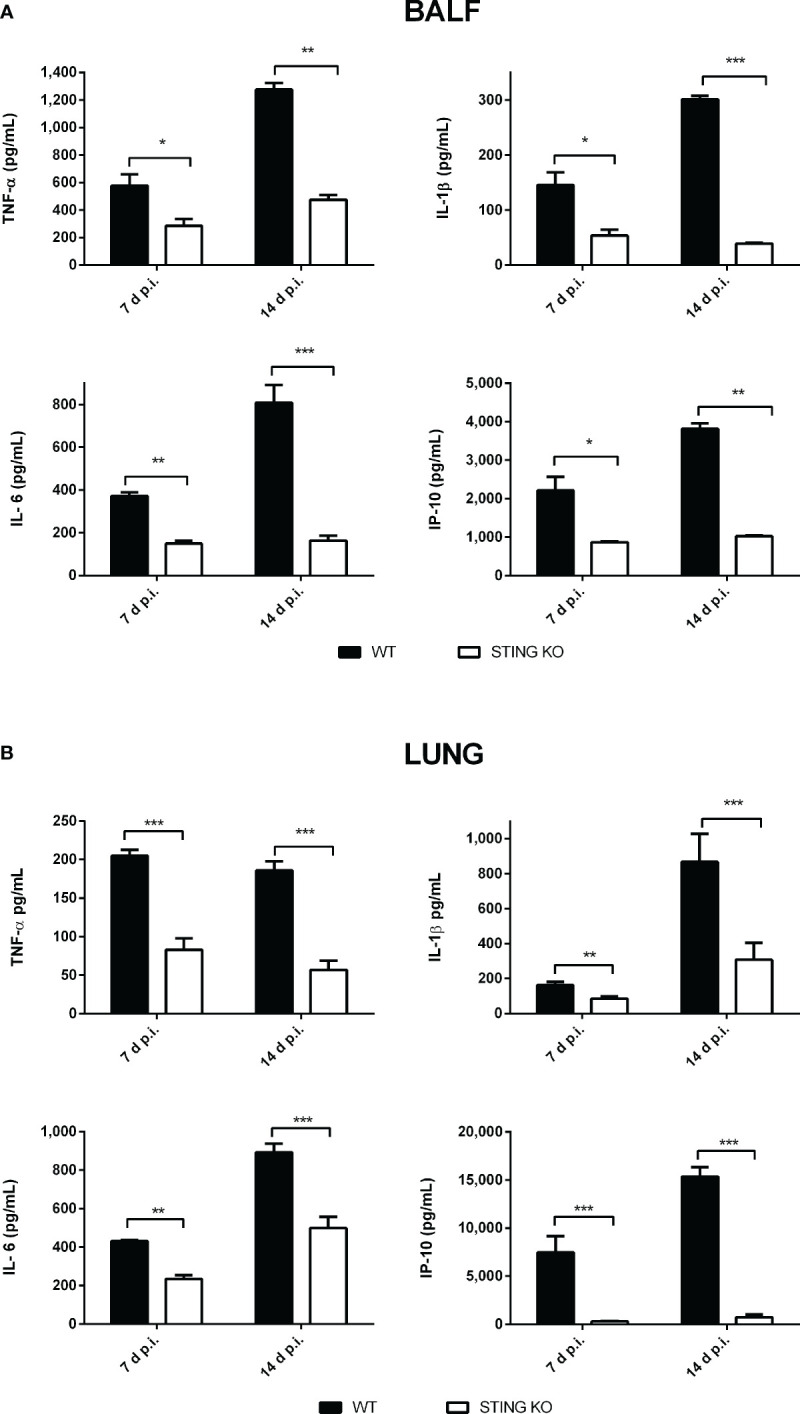
Influence of STING on the pulmonary production of proinflammatory factors after intratracheal *Brucella* infection. Wild type (WT) and STING KO mice (n=6 per group) were infected intratracheally with *B. abortus* (10^5^ CFU/mice) and proinflammatory cytokines were measured at 7 and 14 days p.i. in broncho-alveolar lavage fluid (BALF) **(A)** and lung homogenates **(B)**. Values are means ± SD of two independent experiments (*p<0.05, **p <0.01, ***p<0.001, Student’s *t* test).

**Figure 7:**
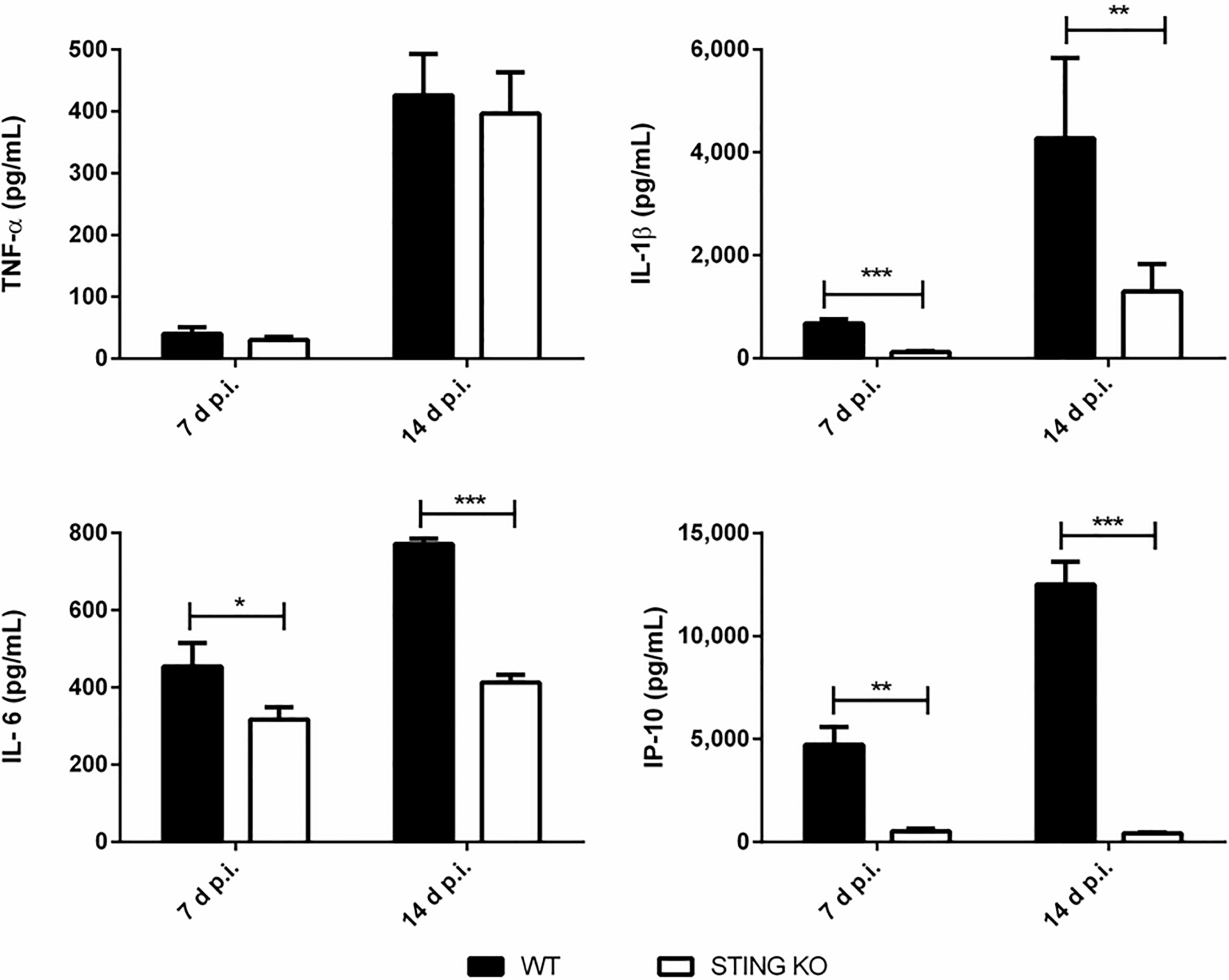
Influence of STING on the production of proinflammatory factors in the spleen after intratracheal *Brucella* infection. Wild type (WT) and STING KO mice (n=5 per group) were infected intratracheally with *B. abortus* (10^5^ CFU/mice) and proinflammatory cytokines were measured at 7 and 14 days p.i. in their spleens. Values are means ± SD of two independent experiments (*p<0.05, **p <0.01, ***p<0.001, Student’s *t* test).

Proinflammatory cytokines play a central role in the control of infections by mediating the recruitment of phagocytes, increasing the expression of cell adhesion molecules and antimicrobial peptides, and activating the microbicidal and antigen-presenting function of phagocytes, among other functions. Given the role of STING in the control of *Brucella* infection in lungs and peripheral organs, and its known contribution to the induction of proinflammatory cytokines, we decided to evaluate the role of STING in the proinflammatory response of lungs and spleen in mice infected with *B. abortus* through the intratracheal route. As shown in **Figure 6A**, the levels of proinflammatory cytokines and chemokines were reduced in the broncho-alveolar lavage fluid (BALF) of STING KO mice as compared to wild type mice at both 7 and 14 days p.i. A similar picture was observed when lung homogenates were tested (**Figure 6B**). Overall, our findings reveal a reduced proinflammatory response to intratracheal *B. abortus* infection in the lungs of STING KO mice as compared to wild type mice. From its respiratory portal of entry *Brucella* can disseminate systemically and reach peripheral organs, among which the spleen is a preferred target in the mouse model of infection. Also in this organ STING appeared to have an important role as a mediator of proinflammatory responses to *B. abortus*. As shown in **Figure 7**, the levels of IL-1β, IL-6 and IP-10 were significantly reduced in the spleens of STING KO mice at 7 and 14 days after intratracheal infection.

### 3.6 cGAS is also involved in the control of respiratory *Brucella* infection

**Figure 8:**
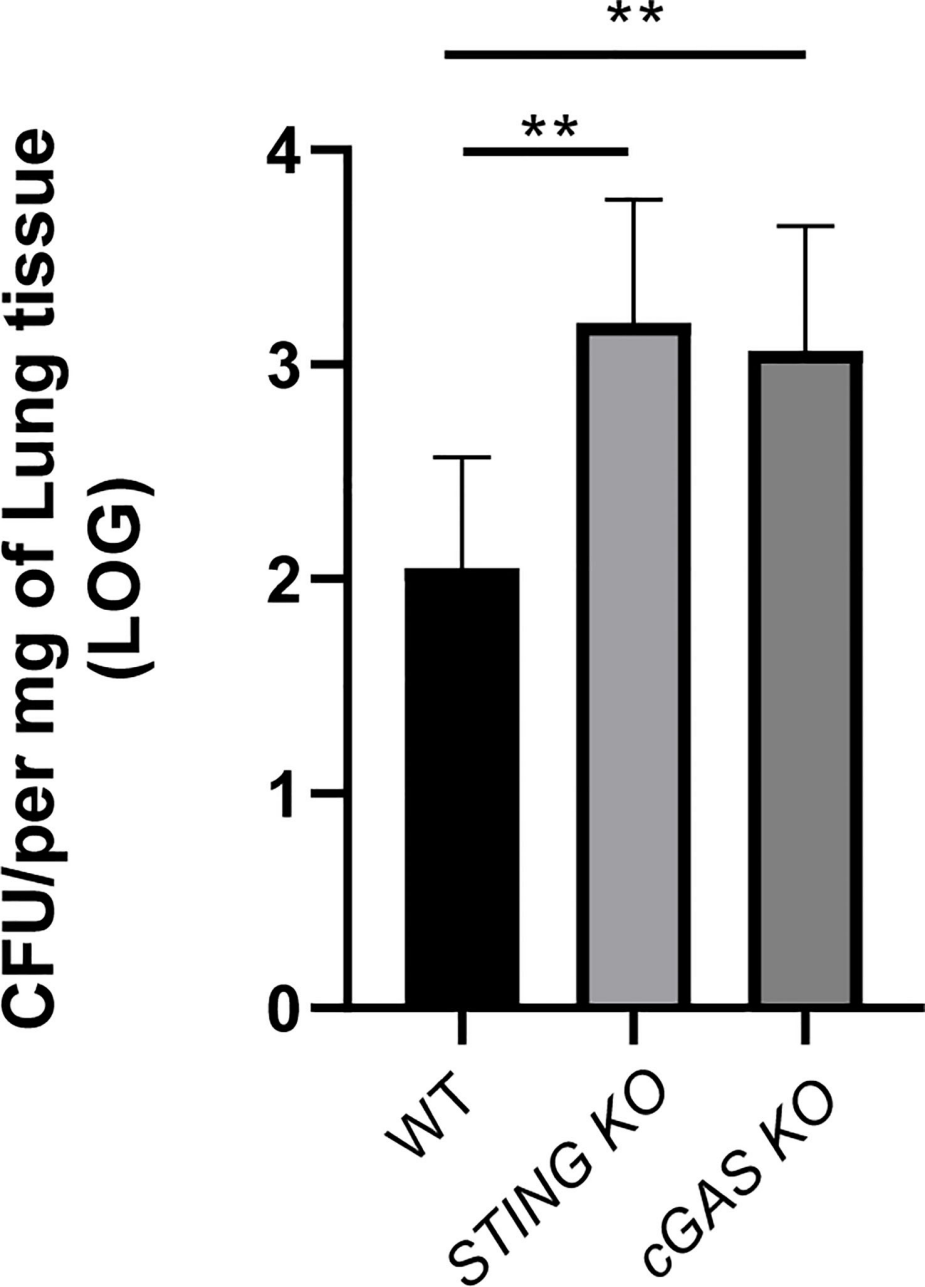
cGAS is involved in the control of *Brucella* infection acquired through the airways. Wild type (WT), cGAS KO and STING KO mice (used for comparison) (n=5 per group) were infected intratracheally with *B. abortus* (10^5^ CFU/mice) and CFU were measured at 14 days p.i. in the lungs. Values are data from a representative experiment of two performed with similar results (**p<0.01, Student’s *t* test).

**Figure 9:**
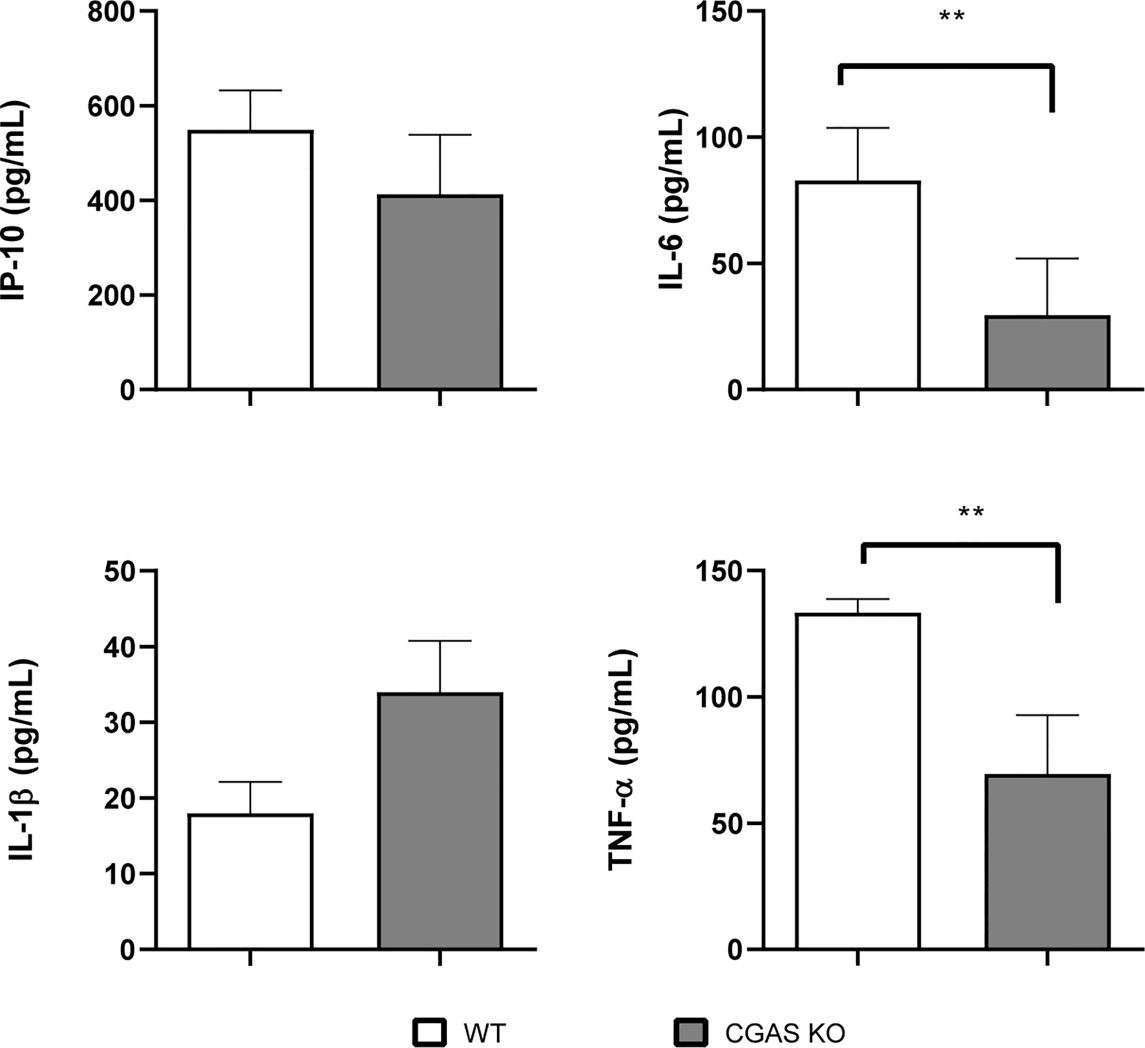
Influence of cGAS on the pulmonary production of proinflammatory factors after intratracheal *Brucella* infection. Wild type (WT) and cGAS KO mice (n=5 per group) were infected intratracheally with *B. abortus* (10^5^ CFU/mice) and proinflammatory cytokines were measured at 14 days p.i. in broncho-alveolar lavage fluid (BALF). Values are means ± SD of two independent experiments (**p <0.01, Student’s *t* test).

Whereas cGAS did not seem to have a role in the control of *B. abortus* infection in alveolar macrophages or lung explants infected *in vitro*, this receptor was shown to have a role in the production of proinflammatory cytokines by these samples during *in vitro* infection. Therefore, a potential role of cGAS in the control of respiratory *B. abortus* infection was evaluated. As shown in **Figure 8**, lung CFU were significantly increased in cGAS KO mice at 14 days after intratracheal infection as compared to WT controls. In addition, the levels of TNF-α and IL-6 were significantly reduced in BALF samples from infected cGAS KO mice as compared to their WT counterparts (**Figure 9**).

## 4 Discussion

Inhalation of aerosols containing *Brucella* spp. constitutes an important form of contagion with this bacterium. From its pulmonary port of entry, the pathogen passes into the systemic circulation and invades various organs. The pulmonary immune response to *Brucella* is poorly understood, and it is not precisely known why it fails to prevent the passage of the bacteria into the general circulation. Nucleic acids are microbial components that can be recognized by the innate immune system. If microbial DNA is found in endosomal compartments it can be recognized by TLR9, while if it is found in the cytosol it can be detected by non-TLR receptors such as AIM2 (component of inflammasomes that mediate secretion of IL-1β and IL-18) and components of the cGAS/STING pathway (that mediate production of type I interferons, autophagy and activation of NF-κB).

Although it was initially considered that cytosolic DNA receptors would only have relevance in the immune control of viral infections, later studies showed that these receptors are also important for the control of infections by other pathogens, including bacteria acquired through the respiratory route (31). For example, AIM2-has been shown to be involved in the control of intratracheal infection with *M. tuberculosis* or *Brucella abortus*, and in both cases AIM2 deficiency was associated with increased bacterial loads in lungs (14, 32). Gram-negative and Gram-positive bacteria, among other microorganisms, are known to promote STING signaling. Moreover, a role for STING in the control of infections produced by bacteria has been reported (18, 20). Both type I IFNs and proinflammatory cytokines may be involved in the STING-dependent response to bacterial infections (33).

It has been shown that STING is important in defense against systemic (intraperitoneal) *B. abortus* infection, mediating not only the production of IFN-β but also proinflammatory cytokines (25). That study also showed that c-di-GMP produced by this pathogen can activate STING and trigger type I IFN responses and inflammasome activation in murine and human cells, and it has been suggested that this cyclic dinucleotide induces the initial STING signaling during *B. abortus* infections. STING activation was absent in cells infected with a *B. abortus* mutant deficient in c-di-GMP production (c-di-GMP guanylate cyclase mutant Δ1520) while it was observed in cells infected with the parental wild type strain. Of note, the mutant induced lower expression levels of IFN-β and IL-1β secretion in bone marrow-derived macrophages (BMDM) as compared with the virulent strain. Recently it has been also shown that STING regulates metabolic reprogramming in macrophages and promotes a tendency towards an inflammatory profile in spleen macrophages in the first days after intraperitoneal *B. abortus* infection in mice (34). Although cGAS and STING have been shown to be expressed in lung cells (22–24), the importance of the cGAS/STING axis in the defense against *Brucella* infection by the respiratory route has not been explored. In this study, we show that alveolar macrophages and lung explants from STING KO mice infected *in vitro* with *B. abortus* had significantly increased CFU counts as compared to those from wild-type mice. The same phenomenon was observed when alveolar macrophages and lung epithelial cells were treated with a STING chemical inhibitor before infection. Thus, the impaired control of *B. abortus* infection in lung explants from STING KO mice may be due to the combined effect of STING deficiency on the infection control capacity of macrophages, epithelial cells and eventually other cell types. While STING deficiency impaired the ability of alveolar macrophages or lung explants to control *B. abortus* infection, no such effect was observed for cGAS deficiency. These results agree with those previously reported for *B. abortus* infections in BMDM (25).

In the present study, lung cells from STING KO or cGAS KO mice or those treated with H151, a STING inhibitor, secreted less TNF-α, IL-1β, IL-6 and/or IP-10 in response to **in vitro* Brucella* infection. A reduced production of proinflammatory cytokines in response to bacterial pathogens in cells deficient in the cGAS/STING pathway has been reported in some studies. For example, STING KO and cGAS KO BMDM produced less cytokines than their WT counterparts after *B. abortus* infection (25). Macrophages from cGAS- and STING-deficient mice were severely impaired in producing proinflammatory cytokines (and type I IFNs) in response to *Legionella pneumophila*, and the same defect was observed when the infection was performed on cells from patients carrying the HAQ variant of STING (35). In addition, cGAS and STING are essential for inducing IFN-β and other ISGs in response to *Mycobacterium tuberculosis* infection in both human and mouse macrophages (36).

The cGAS/STING pathway is involved in the response to several bacterial infections, but its role differs depending on the pathogen and the infection model. The possible contribution of this pathway to defense against bacterial infections raised some doubts, since this pathway mediates the production of type I IFNs (37), which in some cases have been associated with negative effects on antibacterial immunity. However, it has been shown that a negative effect of type I IFNs is not observed in all bacterial infections, with cases in which these IFNs contribute to immune control. Such is the case of *Legionella pneumophila, Streptococcus pyogenes, Streptococcus pneumoniae* and *Helicobacter pylori*, among others (38). In *Francisella tularensis* infections, STING-dependent type I IFN production is necessary for the activation of the AIM2 inflammasome, possibly due to the ability of type I IFNs to activate guanylate-binding proteins (GBPs) by increasing bacteriolysis and the release of DNA into the cytosol (39). Of note, the role of the cGAS/STING pathway cannot be predicted solely from the effects that type I IFNs can exert. For example although mice deficient for the type I IFN receptor (IFNAR) have been described as more resistant to *Listeria monocytogenes* infection (40), STING-deficient mice do not show significant differences in splenic load compared to normal ones (41). Apart from this last study, there are few reports on the impact of the absence of STING (and/or cGAS) in the control of bacterial infections. In the case of *Mycobacterium tuberculosis* infection, STING-deficient mice appear to have no modifications in their bacterial load (pulmonary or extrapulmonary), survival, and proinflammatory cytokine levels compared to normal mice (36, 42). In contrast, we found that STING-deficient mice have higher loads of *B. abortus* in lung, spleen and liver after intratracheal infection, demonstrating that STING is beneficial in controlling *Brucella* infection acquired through the respiratory route. These results agree with the previously reported protective role of STING against intraperitoneal *Brucella* infection (25). While it has been reported that in the first hours after **in vitro* Brucella* infection of macrophages the levels of a microRNA (miR-24) that targets the STING messenger RNA are increased, resulting in a transient reduction of STING levels, this receptor is still required for controlling acute and chronic *B. abortus* infection in mice (43). It has been also reported that STING mediates the induction of the unfolded protein response (UPR) in macrophages infected with *B. abortus* (44). This UPR exerted some control on the production of several proinflammatory mediators (IL-6, IL-1β), and regulated the production of IFN-β and the expression of several factors linked to type I IFN responses. While the UPR was shown to favor *B. abortus* replication in macrophages, the influence of STING on proinflammatory mediators may explain the increased susceptibility of STING KO–derived macrophages to *B. abortus* infection observed in previous studies (25). In agreement with our results showing an increased susceptibility of STING KO mice and cGAS KO mice to respiratory *B. abortus* infection, a recent study in a murine model of intranasal *Legionella pneumophila* infection found that both KO mice have higher bacterial burdens than control mice (35).

As already mentioned, the cGAS/STING pathway mediates the production of both type I IFNs and proinflammatory cytokines. The role of type I IFNs as antiviral cytokines is widely established, and it is well known that they are induced when pattern recognition receptors (PRRs) sense foreign molecules. Nevertheless, recent evidence suggests that type I IFNs can also mediate inflammatory responses to bacterial infections (45). Regarding the role that type I IFNs would play in the control of *Brucella* infection, contradictory results have been obtained. While in 129Sv/Ev mice the absence of IFNAR was beneficial for infection control (15 days p.i.) (46), it did not lead to significant changes in mice from Balb/c background up to 4 weeks p.i (47). In contrast to what was observed *in vivo* , the absence of IFNAR in macrophages from 129Sv/Ev strain mice resulted in a significant increase in the bacterial load at 24 hours p.i., suggesting a protective role for type I IFNs (25). In our model, a reduced *IFN-β* expression, as well as lower concentrations of IP-10, in lungs and spleen of C57BL/6 mice lacking STING were detected. Further studies will be required to establish the role of IFN-β in the reduced protection of STING KO mice against *B. abortus* respiratory infection.

As mentioned, STING exerts other effects in addition to the induction of type I IFN, including stimulation of the NF-kB pathway with the consequent production of proinflammatory cytokines. Previous studies also suggest that type I IFN contribute to the activation of the AIM2 inflammasome, and therefore to caspase-1 activation and IL-1β secretion, through its ability to induce the expression of GBPs that lyse pathogen-containing vacuoles and allow the release of bacterial products, including DNA, to the cytosol (25, 39). We found lower concentrations of proinflammatory cytokines in the BALF, lungs and spleen of *Brucella*-infected STING KO mice and also in BALF of cGAS KO mice, suggesting that these cytokines may be involved in the protective role of STING and cGAS against respiratory *B. abortus* infection.

In conclusion, the present study reveals a protective role of the cGAS/STING pathway during acute respiratory *Brucella abortus* infection. In this context, the cGAS/STING pathway induces the production of proinflammatory cytokines in lungs and peripheral tissues, which may contribute to the cGAS/STING-dependent control of airborne brucellosis. Further research is warranted to establish the precise contribution of this, and eventually other mechanisms, to the cGAS/STING-dependent reduction of bacterial burden.

## Data availability statement

The raw data supporting the conclusions of this article will be made available by the authors, without undue reservation.

## Ethics statement

The animal study was reviewed and approved by Committees for the Care and Use of Experimentation Animals from the UFMG (CETEA no. 69/2020) and the School of Pharmacy and Biochemistry of the University of Buenos Aires (Res. D1879/2019).

## Author contributions

Conceived and designed the experiments: IAP, MF, SCO, PB. Performed the experiments: IAP, MF, RAS, CB, JOW, ECS. Analyzed the data: IAP, RAS, CB, MF, JOW, ECS, SCO, PB. Wrote the draft and/or final version of the paper: IAP, MF, SCO, PB. All authors reviewed the manuscript. All authors contributed to the article and approved the submitted version.
